# Four Large Indels in Barley Chloroplast Mutator (*cpm*) Seedlings Reinforce the Hypothesis of a Malfunction in the MMR System

**DOI:** 10.3390/ijms26178644

**Published:** 2025-09-05

**Authors:** Franco Lencina, Alberto R. Prina, María G. Pacheco, Ken Kobayashi, Alejandra M. Landau

**Affiliations:** 1 Instituto Nacional de Tecnología Agropecuaria (INTA), Instituto de Genética, Dr. Nicolás Repetto y De Los Reseros s/n, Hurlingham 1686, Buenos Aires, Argentina; alberto.prina55@gmail.com (A.R.P.); pacheco.maria@inta.gob.ar (M.G.P.); landau.alejandra@inta.gob.ar (A.M.L.); 2 Laboratorio de Agrobiotecnología, Grupo Biología Molecular Vegetal Aplicada, Instituto de Biodiversidad y Biología Experimental y Aplicada (IBBEA, CONICET-UBA), Departamento de Fisiología, Biología Molecular y Celular, Facultad de Ciencias Exactas y Naturales, UBA, Buenos Aires 1891, Argentina; ken.biolo@gmail.com

**Keywords:** plastome, chloroplast DNA, organelles, illegitimate recombination, direct repeats, mismatch repair system

## Abstract

A mutation detection strategy based on mismatch digestion was applied previously in barley seedlings carrying the chloroplast mutator (*cpm*) genotype through many generations. Sixty-one mutations were detected along with four large indels: a 15 bp insertion in the intergenic region between *tRNA^His^* and *rps19* genes, a 620 bp deletion in the *psbA* gene, a 79 bp deletion in the intergenic region between *rpl33* and *rps18* genes and a 45 bp deletion in the *rps3* gene. The present investigation aims to understand the mechanisms producing the large indels and to better characterize the *cpm* mutagenic effect. Whole plastome sequencing revealed novel polymorphisms that were identified either in regions not previously examined or in regions that were explored but not detected through celery juice extract (CJE) digestion. The 620 bp deletion in the *psbA* gene was lethal when homoplastomic, whereas the 45 bp deletion in the *rps3* gene did not affect the viability of the seedlings even in homoplastomy. The presence of direct repeats at the borders of large indels suggests that they could have originated by illegitimate recombination because of CPM protein malfunction. A truncated mismatch repair MSH1 protein identified in *cpm* seedlings suggests that CPM is involved in organellar genome stability maintenance.

## 1. Introduction

The chloroplast genome, or plastome, is considered highly conserved compared to the plant nuclear genome. However, complex patterns of variation are observed when comparing plastomes across different species [1]. Some of these differences involve length variations caused by insertions or deletions (indels) of several bases or more [2].

Certain nuclear gene mutants lead to an increased frequency of mutations in the plastome, making them valuable tools for expanding the limited cytoplasmic variability available for research and plant breeding [3,4,5,6]. One such mutant is the barley chloroplast mutator (*cpm*), initially described as inducing a wide range of cytoplasmically inherited chlorophyll mutations. Genetic studies determined that *cpm* is a homozygous recessive nuclear gene [7]. In early work, several chlorophyll-deficient mutants were isolated and stabilized by crossing them as female parents with wild-type (WT) male plants. This strategy preserved the mutated cytoplasms while restoring a normal nuclear background, effectively eliminating the action of the mutator gene and converting the mutants into cytoplasmic lines. At that time, these lines were only characterized phenotypically [8].

The first molecular evidence of *cpm* gene activity on the chloroplast genome was obtained through the sequencing of candidate genes such as *infA*, *ycf3*, and *psbA* in stabilized lines. These lines exhibited chlorophyll-deficiency in the cases of *infA* and *ycf3* genes, and herbicide tolerance targeting photosystem II (PSII) in the case of *psbA* gene [9]. The DNA changes consisted of substitutions and one-nucleotide indel [9].

Later, to investigate plastome polymorphisms on a larger scale, a mutation detection strategy based on mismatch digestion, known as cpTILLING (chloroplast Targeting Induced Local Lesions in Genomes), was applied to 304 seedlings carrying the *cpm* gene [10]. These seedlings belonged to two groups of families that had maintained the *cpm* gene through different numbers of generations of natural self-pollination. Analysis of 31 PCR amplicons covering 33 genes and several intergenic regions of the plastome revealed 61 distinct one- or two-nucleotide polymorphisms, indicating a highly unstable chloroplast genome in these seedlings. Most of these polymorphisms were due to substitutions and small indels in microsatellites.

Interestingly, a peculiar pattern involving combinations of five single nucleotide polymorphisms was observed in the *rpl23* gene, which encodes a chloroplast ribosomal protein. This was later explained not as true mutations, but as the result of increased illegitimate recombination between the *rpl23* gene and its pseudogene [11].

In addition to the aforementioned polymorphisms, four large indels were identified: a 15 bp insertion in the intergenic region between the *tRNA^His^* and *rps19* genes; a 620 bp deletion in the *psbA* gene, which encodes the D1 protein of PSII; a 79 bp deletion in the intergenic region between *rpl33* and *rps8*; and a 45 bp deletion in the *rps3* gene, which encodes a ribosomal protein [10]. Short identical sequences, i.e., perfect direct repeats or similar sequences, i.e., imperfect direct repeats, were found at both ends of the four large indels. This suggested that, as in the case of the *rpl23* gene and its pseudogene [11], these indels could also result from increased illegitimate recombination due to malfunction of the *Cpm* gene product.

In this work, to better understand the mutagenic effect of *cpm* and the origin of these large indels, the flanking sequences were analyzed in detail. A capillary electrophoresis system (Fragment Analyzer™) was used instead of polyacrylamide gel electrophoresis, improving the visualization of digestion bands produced by celery juice extract (CJE) [10].

Furthermore, the complete plastome of seedlings carrying the large indels was sequenced to compare the polymorphism detection capabilities of Next Generation Sequencing (NGS) with CJE digestion and to identify additional polymorphisms with potential functional consequences.

Additionally, distinct consequences were observed for the two large indels located in coding regions (*psbA* and *rps3*).

The *cpm*-induced polymorphisms—including substitutions, small indels in microsatellites, and illegitimate recombination between *rpl23* and its pseudogene—represent a spectrum of changes similar to those found in mutants of the DNA mismatch repair (MMR) system [12]. In this context, the identification of a substitution introducing a premature stop codon in exon 17 of the *cpm Msh1* gene [13] aligns with the findings of this study, which indicate altered recombination also observed in mutants with defective MSH1 protein function.

The findings presented in this work have significant implications for plant research, particularly in the fields of organellar genome stability, mutagenesis, and functional genomics. The identification of large indels flanked by short direct repeats in *cpm* seedlings provides compelling evidence for the role of MSH1 in suppressing illegitimate recombination in plastids. This insight can offer a model system to study recombination mechanisms and mismatch repair pathways in organelles, which remain less understood compared to nuclear genomes.

## 2. Results

### 2.1. Four Large Indels Detected by CJE Digestion

Using the cpTILLING strategy, four seedlings were identified as carrying distinct large indels—one per seedling—in addition to substitutions and small indels [10]. In that earlier study, sequencing of the rps19 amplicon revealed a 15 bp insertion, while the rps3 amplicon exhibited a 45 bp deletion. For the psbA and rpl33 amplicons, sequencing of gel-purified bands confirmed deletions of 620 bp and 79 bp, respectively.

**Figure 1:**
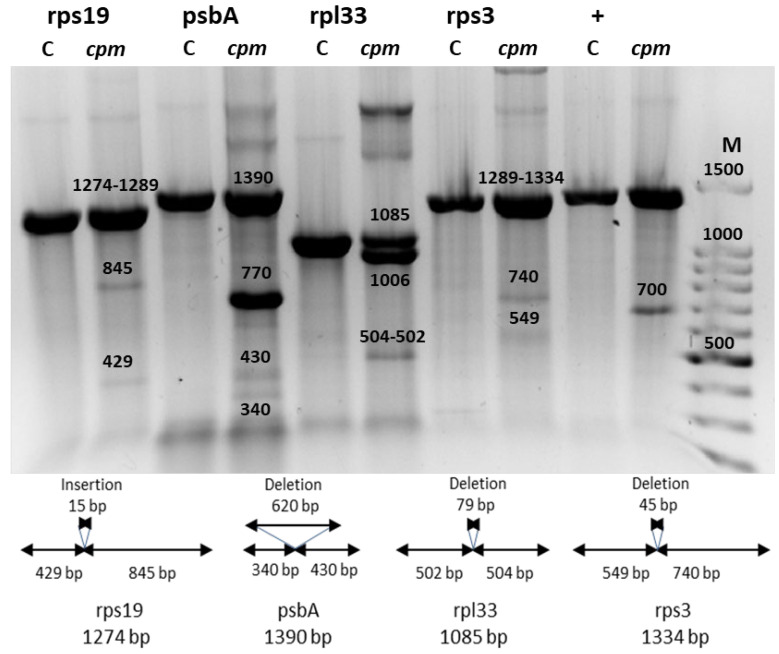
Celery juice extract (CJE) digestion analysis of rps19, psbA, rpl33, and rps3 amplicons containing large indels, visualized on agarose gels. M: Molecular weight marker (100 bp ladder); C: Wild-type (WT) control seedling; *cpm*: *cpm* seedlings carrying large indels; “+” denotes the positive control for CJE digestion, consisting of a *cpm* mutant carrying a TA insertion in the middle of the *psbC* gene. Molecular weights of digestion products were annotated on the gel based on sequencing data.

In the present study, the mutation producing a premature stop codon in the Msh1 gene was identified in the four seedlings by sequencing. The digestion patterns of the four amplicons with CJE were re-examined (see Materials and Methods), and the results are shown in Figure 1. In addition to the full-length WT band and its corresponding digestion fragments, a second, smaller band—indicative of a deletion—was observed in the psbA and rpl33 amplicons.

Interestingly, digestion bands were only visible when *cpm* DNA was mixed with WT DNA, as in the case of the rps19 and rps3 amplicons. In contrast, for the psbA and rpl33 amplicons, digestion bands were detected using *cpm* DNA alone. These results suggest that the indels in psbA and rpl33 amplicons are present in a heteroplastomic state, while those in rps19 and rps3 amplicons are homoplastomic.

During the initial screening, the rps19 amplicon exhibited a band of higher molecular weight than the WT counterpart on polyacrylamide gels (Figure S1). This size shift could not be attributed to the 15 bp insertion alone; as such, a small difference would not be resolved in a 3% polyacrylamide gel. Instead, the anomalous band likely represented a heteroduplex formed by the annealing of DNA strands with and without large indels, resulting in mismatched base pairing.

**Figure 2:**
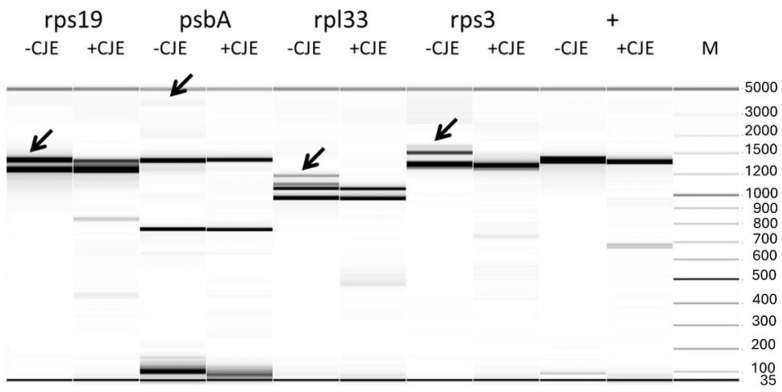
Virtual gel image of CJE digestions analyzed using the Fragment Analyzer™ system (Agilent, Santa Clara, CA, USA) for heteroduplexes derived from amplicons containing large indels: rps19, psbA, rpl33, and rps3. Arrows indicate the presence of heteroduplex bands prior to CJE digestion (-CJE), which disappear following digestion (+CJE), confirming successful cleavage. M: molecular weight marker (100 bp ladder). “+” denotes the positive control for CJE digestion, consisting of a *cpm* mutant carrying a TA insertion in the middle of the *psbC* gene.

To determine whether similar high-molecular-weight bands were generated during the re-annealing step following amplification of the other three amplicons, and whether these were affected by CJE digestion, the samples were analyzed using capillary electrophoresis on a Fragment Analyzer™ system, both with and without CJE treatment (Figure 2). In all cases, capillary electrophoresis revealed an additional band larger than the expected amplicon size in the undigested samples. These bands disappeared following CJE digestion, indicating successful cleavage of heteroduplexes. However, for the rps19 amplicon, the larger band persisted partially after digestion, consistent with the incomplete cleavage observed in the original analysis where the large indel was first detected (Figure S1).

### 2.2. Presence of Perfect or Imperfect Direct Repeats Flanking the Large Indels

**Figure 3:**
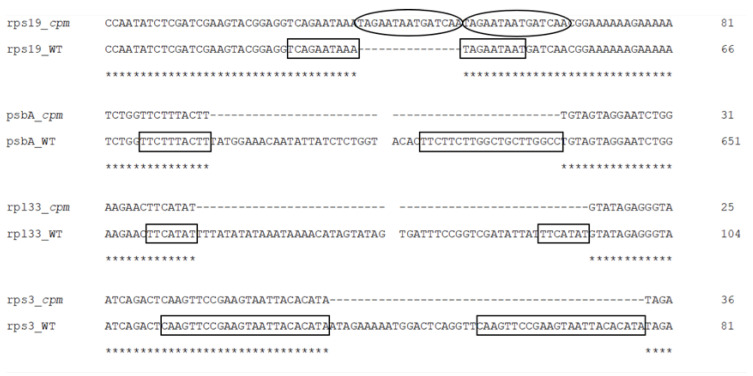
Sequence alignments of amplicon regions flanking the large indels in *cpm* mutants compared to WT control seedlings. Direct repeats are highlighted with boxes, and the duplicated sequence within the insertion in the rps19 amplicon is indicated with an ellipse. Gaps in the psbA and rpl33 alignments represent discontinuities in the sequence. Asterisks (*) denote identical nucleotides across the aligned sequences.

Sequencing of the four amplicons harboring large indels revealed the presence of short, identical, or nearly identical sequences flanking both ends of each indel (see alignments in Figure 3). These sequences were classified as perfect or imperfect direct repeats, depending on whether they were identical or only partially similar.

The indels identified in the rps19 and psbA amplicons were flanked by imperfect direct repeats. In the intergenic region between *tRNA^His^* and *rps19*, the 15 bp insertion—representing a tandem duplication—was bordered by two slightly divergent repeats: one 10 bp in length and the other 9 bp. Similarly, the deletion within the *psbA* gene was flanked by direct repeats measuring 10 bp and 17 bp, respectively.

In contrast, the indels in the *rpl33*–*rps18* intergenic region and the *rps3* gene were flanked by perfect direct repeats. These consisted of identical sequences of 7 bp and 24 bp, respectively.

The sequences and lengths of all identified direct repeats are summarized in Table 1.

### 2.3. The RPS3 Protein Encoded by the Deleted rps3 Gene in Barley Is the Same Size as the WT RPS3 Protein in Other C4 Plants

**Figure 4:**
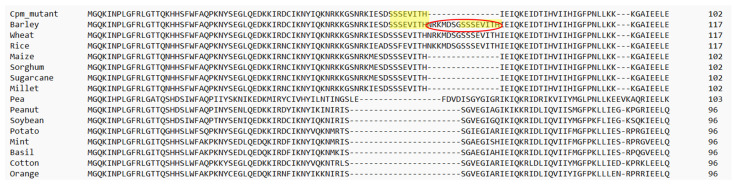
Alignment of the first part of RPS3 protein sequences from the *rps3* mutant isolated in *cpm* seedlings, WT barley, and diverse species. The 15 amino acids absent in the *cpm* mutant due to a 45 bp deletion is marked by a red ellipse. Amino acids corresponding to direct repeats are highlighted in yellow in the WT barley and the *rps3 cpm* mutant.

The 45 bp deletion identified in the *rps3* gene results in the loss of a 15-amino-acid segment from the RPS3 ribosomal protein in barley (Figure 4). Interestingly, this deletion was found in a homoplastomic state, and the affected seedling exhibited a normal green phenotype, although its leaf blades were noticeably narrower and longer than those of WT control seedlings.

Sequence alignment of the RPS3 polypeptides from the *cpm* mutant, WT barley, and other species, revealed that in other C4 plants, i.e., maize, sorghum, sugarcane and millet, RPS3 protein naturally lacks the same 15-amino-acid segment missing in the *cpm* mutant (Figure 4). On the contrary, other C3 cereals, such as wheat and rice, have conserved the 15 amino acids, as well as barley. Interestingly, part of this segment is also missing in other plants belonging to legumes, aromatic herbs, fruit trees, etc., along with other regions of the protein next to the 15-amino acid segment. This suggests that the deleted region may not be essential for RPS3 function.

### 2.4. The Deletion in the psbA Gene Disrupts PSII Function and Causes Seedling Lethality

A 620 bp deletion in the *psbA* gene was initially identified in a heteroplastomic state in one *cpm* F_12_ seedling. Subsequent screening of progeny from the same family led to the identification of a seedling carrying the same deletion in a homoplastomic state.

This deletion causes a frameshift and introduces a premature stop codon, resulting in a truncated D1 protein consisting of only 83 amino acids. The seedling harboring the homoplastomic deletion exhibited a *viridis* phenotype (homogeneous light green) and died approximately two weeks after sowing (Figure S2), indicating a lethal disruption of photosynthetic function.

**Figure 5:**
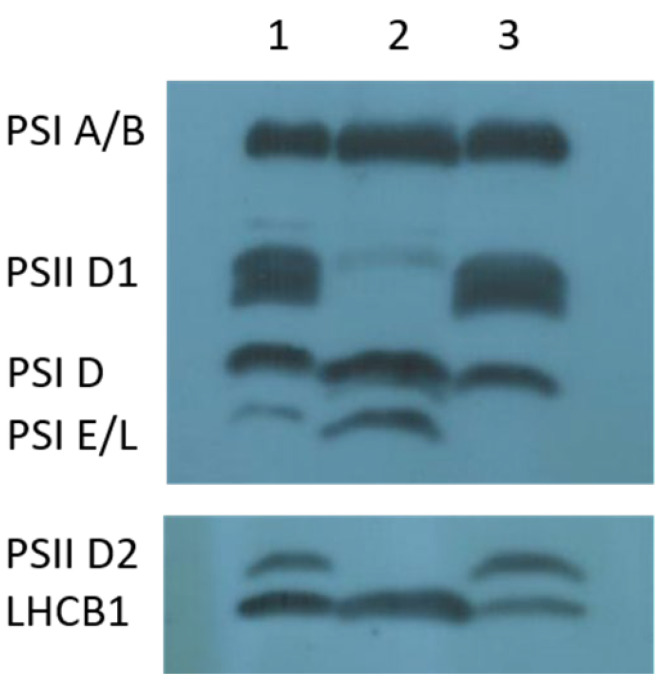
Immunoblot analysis of photosystem proteins in the *cpm* mutant homoplastomic for the 620 bp deletion in the *psbA* gene (*viridis* phenotype) and in normal green *cpm* siblings. Lanes 1 and 3: normal green siblings; Lane 2: *viridis* mutant. The blot shows altered expression of photosystem II (PSII) core proteins (D1 and D2) in the mutant, while photosystem I (PSI) core proteins and PSII associated light-harvesting antenna (LHCB1) are expressed at comparable levels in both mutant and normal green *cpm* siblings.

To assess the impact of the truncated D1 protein on photosynthetic complexes, thylakoid membrane proteins were analyzed by Western blot in both the *psbA* mutant and normal green *cpm* seedlings. The *psbA* mutant showed aberrant expression of the D1 and D2 proteins, which are core components of PSII. In contrast, proteins associated with photosystem I (PSI-A/B, D, E, L) and LHCB1, a component of the PSII light-harvesting antenna, were expressed at normal levels. All these proteins were properly expressed in the normal green *cpm* seedlings (Figure 5).

### 2.5. Complete Plastome Sequencing Confirms Large Indels Identified by cpTILLING and Reveals Additional Polymorphisms

To validate the large indels identified by cpTILLING and to detect additional mutations not covered by this method—which targeted only ~30% of the plastome—complete chloroplast genome sequencing was performed on the four *cpm* seedlings carrying large indels.

De novo assembly of sequencing reads produced scaffolds spanning the Large Single Copy (LSC), Small Single Copy (SSC), and Inverted Repeat (IR) regions of the chloroplast genome. This analysis confirmed the presence of the four large indels and also revealed additional polymorphisms across the plastome (Figure S3). These included single nucleotide polymorphisms (SNPs) and small insertions or deletions (1–2 bp), particularly within microsatellite regions, identified by mapping reads to the reference genome.

**Figure 6:**
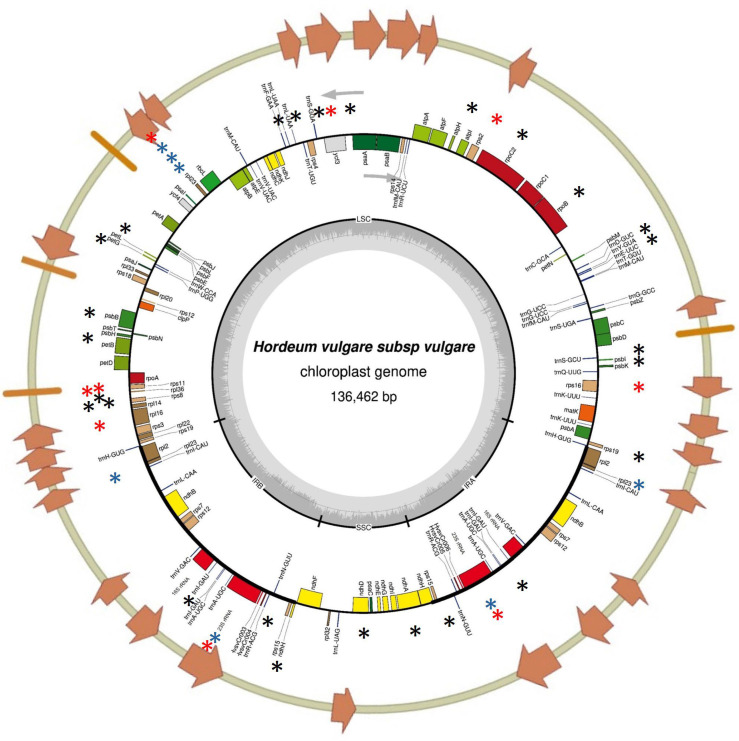
Polymorphisms identified in the chloroplast genome of seedlings carrying large indels by Next generation sequencing (NGS). Orange bars and arrows indicate the regions of the barley chloroplast genome that were previously analyzed using chloroplast Targeting Induced Local Lesions in Genomes (cpTILLING). Black asterisks mark novel polymorphisms identified by NGS in regions not previously examined. Blue asterisks denote polymorphisms that were detected both in previous studies using CJE digestion and in the present study using NGS. Red asterisks highlight polymorphisms newly identified in this study by NGS that were not detected by CJE digestion in earlier analyses.

Average sequencing coverage was high across all samples: 528× for rps19, 509× for psbA, 262× for rpl33, and 470× for rps3 amplicon mutants. All identified polymorphisms are listed in Tables S1–S5 and shown in Figure 6. Variants were considered homoplastomic if they were present in more than 95% of the mapped reads.

#### 2.5.1. Plastome of the rps19 Amplicon Insertion Mutant

In the F_12_ seedling carrying the large insertion in the rps19 amplicon, complete plastome sequencing revealed nine substitutions and twelve small indels, most of which were located in the LSC region.

Among the substitutions found in coding regions, mutations were identified in the *rpoB*, *ndhD*, *rpl23*, and *23S rRNA* genes. All were present in a homoplastomic state. Notably, the substitutions in *rpoB*, *ndhD*, and *rpl23* were missense mutations. The variants in *rpl23* and *23S rRNA* had previously been detected by CJE digestion and by NGS in this work.

NGS also uncovered novel polymorphisms in the *rpoB*, *ndhD*, and *ycf3* genes. Although the *ycf3* gene had been analyzed by cpTILLING in a previous study, an insertion in intron 1 of *ycf3* was detected only by NGS and not by CJE digestion, highlighting the increased sensitivity of whole-plastome sequencing.

Additionally, the 15 bp insertion originally identified in the rps19 amplicon was confirmed by the VarScan algorithm (Table S1).

#### 2.5.2. Plastome of the psbA Gene Deletion Mutant

In the homoplastomic offspring of the F_12_ seedling carrying the large 620 bp deletion in the *psbA* gene, complete plastome sequencing revealed eleven single nucleotide substitutions and fourteen small indels, primarily located in intergenic regions of the LSC region.

All substitutions identified within coding sequences were silent mutations and present in a homoplastomic state. Only one small deletion was detected in a coding region, the *rpoC2* gene, in heteroplastomic state. Additionally, four substitutions and one insertion were detected in the *rpl23* pseudogene in homoplastomy.

Several novel polymorphisms were identified by NGS that had not been detected in previous cpTILLING analyses. These included one substitution in the *rpl23* pseudogene, as well as variants in the *rps16* gene, along with two small indels in the intergenic trans-splicing region of *rps12*.

Conversely, some polymorphisms previously detected by CJE digestion were confirmed by NGS, including an indel at position 30348 in an intergenic region, and three substitutions plus one indel in the *rpl23* pseudogene (Table S2).

#### 2.5.3. Plastome of the rpl33 Amplicon Deletion Mutant

In the F_6_ seedling carrying the large deletion in the rpl33 amplicon, complete plastome sequencing identified four single nucleotide substitutions and four small indels. Among the substitutions located in coding regions, one was a missense mutation in the *rps8* gene, present in a homoplastomic state, and another was a missense mutation in the *rpl23* gene, found in a heteroplastomic state. The latter had been previously detected by CJE digestion, confirming its presence (Table S3).

#### 2.5.4. Plastome of the *rps3* Gene Deletion Mutant

In the F_6_ seedling carrying the large deletion in the *rps3* gene, complete plastome sequencing identified eight single nucleotide substitutions and five small indels, most of which were located in the LSC region.

Four substitutions and one insertion were found in the *rpl23* pseudogene (all in homoplastomy), while one substitution in the *rpl23* gene was present in a heteroplastomic state. Among these, only one polymorphism—a substitution at position 56846 in the *rpl23* pseudogene—was newly detected by NGS in this study and had not been previously identified by CJE digestion. Additionally, a small indel at position 76784 in the intergenic trans-splicing region of *rps12* was also newly detected.

The only small indel found within a coding sequence was located in the *23S rRNA* gene and was present in a heteroplastomic state. This indel had not been detected by CJE digestion. The only substitution in a coding region was a silent mutation in the *ndhD* gene, found in homoplastomy. This region had not been screened in previous cpTILLING analyses (Table S4).

Furthermore, several polymorphisms were shared among two, three, or all four seedlings carrying large indels (Table S5). Notably, a single nucleotide deletion in an intergenic region (position 16106, 1T deletion) and a substitution in the intron of the *rpl16* gene were found in all four mutants as well as in the WT control when compared to the reference sequence, suggesting these may represent natural variation rather than induced mutations. The substitution within the *rpl16* gene, in a region previously analyzed by cpTILLING, was not detected at that time because, as a mismatch digestion technique, it requires mismatch formation, and since the polymorphism also exists in the WT, no mismatch could be formed for detection.

## 3. Discussion

Large insertions and deletions (indels) have previously been reported in chloroplast genomes of various plant species, particularly at the junctions between the IRs and the LSC or SSC regions [14], as well as in intergenic regions, such as *16S rRNA*–*trnI* [15], and the *rbcL*–*psaI* region [16,17,18]. In the present study, all large indels—except for the deletion in rpl33 amplicon—were also located at IR/LSC or IR/SSC boundaries, reinforcing the notion that these junctions are hotspots for structural variation.

Unlike the numerous small indels (1–2 bp) previously identified, which often occur in mononucleotide tandem repeats [10], none of the large indels described here were associated with such repetitive motifs. This distinction suggests that different mutational mechanisms may underlie the formation of large versus small indels in the plastome.

The successful detection of these large indels using the cpTILLING approach demonstrates that CEL I nuclease (CJE) is capable of cleaving heteroduplex DNA containing loop structures formed by large insertions or deletions [10]. However, the apparently incomplete digestion of the smallest indel—a 15 bp insertion in the rps19 amplicon—may indicate a negative correlation between CJE digestion efficiency and indel size. This observation warrants further investigation to determine whether smaller loops are less accessible or less efficiently cleaved by the enzyme.

Additionally, the presence of slower-migrating bands corresponding to heteroduplexes in polyacrylamide gels suggests that loop structures significantly affect DNA mobility during electrophoresis. Similar reductions in heteroduplex mobility have been reported in other studies [19,20], supporting the idea that structural distortions influence gel migration patterns.

Complete plastome sequencing not only confirmed the presence of the four large indels but also revealed additional single-nucleotide substitutions and small indels (1–2 bp) that had not been previously detected by cpTILLING [10]. This finding aligns with earlier observations comparing mutation detection by sequencing and CJE digestion [21], highlighting that sequencing does not depend on the variable efficiency of endonuclease digestion. Furthermore, NGS may offer superior sensitivity for detecting heteroplasmic mutations compared to CJE digestion. Notably, this sequencing approach identified the same polymorphisms in the *rpl23* gene and its pseudogene across all four large-indel seedlings, consistent with results obtained through CJE digestion [11]. Furthermore, plastome analysis showed that the large-indel seedlings from the F_12_ generation had more polymorphisms than those analyzed in the F_6_ generation.

It is noteworthy that all large indels analyzed in this study were flanked by short direct repeats, either perfect or imperfect (see Table 1). Such sequence features are consistent with the involvement of microhomology-mediated recombination, a form of illegitimate recombination, which has been proposed as a mechanism underlying insertions and deletions in grass plastomes [14,16]. As described by Lovett [22], illegitimate recombination encompasses a variety of mechanisms, including replication slippage at short homologous sequences and cut-and-join events at non-homologous sites.

In nature, illegitimate recombination between short, similar repeats is tightly regulated, thereby preserving chloroplast genome integrity and minimizing the potentially deleterious effects of indels within coding regions. As a result, such recombination events are rare and typically observed only over evolutionary timescales [23]. The frequency of recombination between repeats decreases with shorter repeat length, greater distance between repeats, or increased sequence divergence [24]. Our findings support the hypothesis that enhanced illegitimate recombination between similar flanking repeats is responsible for the three large deletions identified in *cpm* seedlings. This mechanism has been reported to result in the loss of the intervening DNA segment along with one of the repeats [14,16,17], a pattern consistent with the deletions observed in our study. In contrast, the insertion detected in the rps19 amplicon likely arose through a different mechanism, although illegitimate recombination may still have contributed. Notably, the largest direct repeat in the rps3 amplicon is only 24 bp, substantially shorter than the ~200 bp typically required for homologous recombination in *E.coli* [22,25] and commonly used for transgene integration into chloroplast genomes via homologous recombination [26,27].

The anti-recombination activity of MMR proteins plays a critical role in suppressing illegitimate recombination between divergent short sequences by recognizing mismatches within heteroduplex intermediates formed during recombination events [22,28]. In Arabidopsis, the MMR-deficient mutant line *msh1*, which lacks the organellar anti-recombination function of the MSH1 protein [29,30], exhibits large indels in plastome regions containing short, similar repeats (10–15 bp). This kind of structural rearrangements by repeat-mediated recombination was then confirmed by long-read sequencing of mitochondrial and plastid genomes in this *msh1* mutant [31]. Similarly, we propose that the elevated frequency of illegitimate recombination events leading to large indels in the plastome of *cpm* seedlings may result from the loss of MSH1-mediated anti-recombination activity. Supporting this hypothesis, a substitution introducing a premature stop codon in exon 17 of the *Msh1* gene was identified in *cpm* seedlings [13], likely accounting for the plastome instability observed in this line.

The two large indels affecting coding sequences exhibited distinct phenotypic consequences. Notably, only the deletion in the *psbA* gene resulted in a lethal phenotype when homoplastomic. This outcome exemplifies how even non-functional mutations can become fixed in plastomes. In the context of plastid inheritance, such fixation may occur stochastically through vegetative segregation and genetic drift during organelle transmission [32]. Unlike nuclear mutations, plastid mutations do not follow Mendelian segregation and can drift toward homoplastomy regardless of their functional impact, particularly in the absence of strong selective pressure during early development.

The homoplastomic deletion in the *psbA* gene led to a nonfunctional PSII, due to the loss of both the D1 and D2 proteins. This outcome is consistent with findings in *Chlamydomonas reinhardtii*, where it is well established that D1 and D2 proteins are mutually dependent for accumulation [33]. Unlike the heterotrophic alga *C. reinhardtii*, however, the absence of these core PSII components is lethal in higher plants [34]. In contrast, the homoplastomic deletion in the *rps3* gene did not result in lethality. Notably, the 15 amino acids lost in the RPS3 protein correspond to a peptide that distinguishes the WT maize, sorghum, sugarcane, and millet RPS3 protein from that of barley [35], wheat, and rice. Comparative analyses of RPS3 sequences across several species have shown that this peptide is not universally conserved. Although the functional impact of this deletion was not directly assessed in the barley mutant, the lack of a lethal phenotype and the natural variation in this region among species suggest that the deleted peptide is not essential for RPS3 function. Whole plastome sequencing of the two plants exhibiting large gene deletions revealed several polymorphisms within coding regions. Most of these were synonymous substitutions or heteroplastomic small indels that did not appear to affect phenotype. Notably, a missense mutation was identified in the *rpl23* gene; however, this alteration was not associated with any observable phenotypic changes [11].

The results of this study contribute to a deeper understanding of the relationship between a component of the MMR system, the MSH1 protein, and recombination processes in plastids. Unlike the nucleus, recombination is a common mechanism for DNA repair in organelles. Compared to mitochondria, plastids show fewer documented cases of recombination failure, likely due to the lower abundance of repetitive sequences in the plastid genome. The effects on other DNA repair mechanisms of the MSH1 protein have not been investigated in the *cpm* mutant. However, to our knowledge, there is currently no strong evidence supporting a role for MSH1 in repair mechanisms unrelated to recombination. In this context, it is plausible that the repair of DNA double-strand breaks is compromised in the *cpm* mutant, given that recombination—either homologous or illegitimate, such as microhomology-mediated end-joining (MMEJ)—is a key pathway for resolving such lesions [36]. The presence of illegitimate recombination events in *cpm* highlights a potentially broader impact of MSH1 dysfunction on genome stability, warranting further investigation.

Future research should aim to dissect the impact of the truncated MSH1 protein on the plastid genome across diverse genetic backgrounds and species. This could be achieved through gene editing approaches, potentially revealing conserved or divergent mutagenic patterns and thereby enhancing our understanding of organellar genome evolution. Additionally, the effect of the truncated MSH1 variant on the mitochondrial genome in the *cpm* barley mutant remains unexplored and merits further study.

From an applied perspective, the ability of *cpm* to generate functional allelic variants—rather than complete knockouts—represents a valuable tool for functional genomics. This feature enables the development of plastid mutants with subtle phenotypes, ideal for dissecting gene function without compromising plant viability. Some of these plastome gene mutations could be harnessed in breeding programs, for instance, to develop herbicide-tolerant lines, as previously demonstrated [9]. Moreover, it may be possible to identify mutants with enhanced Rubisco efficiency, potentially improving photosynthetic performance and crop productivity. Finally, the truncated MSH1 version in the *cpm* mutant offers a unique opportunity to uncover mutations in genes with poorly understood functions, providing novel insights into plastid biology and expanding the toolkit for both functional genomics and plant breeding.

## 4. Materials and Methods

**Figure 7:**
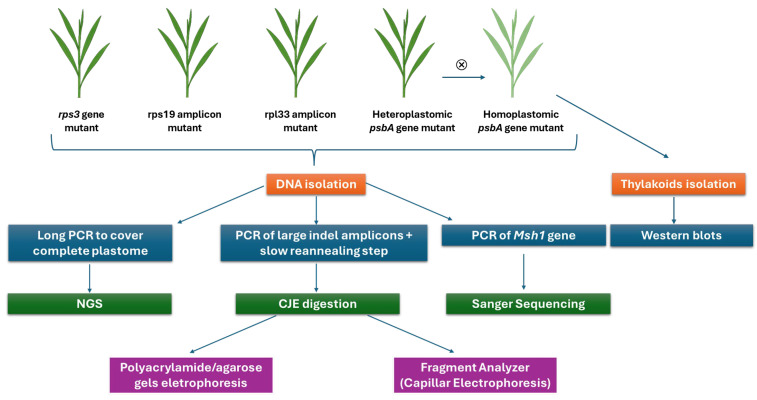
Schematic diagram of the experimental design. The four seedlings carrying the large indels were subjected to DNA isolation followed by molecular and genomic analyses. Only homoplastomic *psbA* mutant offspring of the heteroplastomic *psbA* gene mutant was analyzed at the protein level by western blot. ⮾ means self-pollination.

Figure 7 provides an overview of the experimental workflow carried out in this study. It summarizes the analyses performed on the four *cpm* seedlings carrying large indels, including DNA isolation, digestion of large indel amplicons, plastome sequencing, and protein-level analysis, where applicable.

### 4.1. Plant Material

The four *cpm* seedlings, each harboring one of the large indels analyzed in this study, were previously identified using a cpTILLING approach [10]. The WT control was a two-row cultivated barley (*H. vulgare*) genotype homozygous for the chromosome reciprocal translocationT (6–7)a, which corresponded to the parental genotype that was originally mutagenized with a combined treatment of X-rays and sodium azide to generate the *cpm* mutant line [7]. Successive generations of *cpm* families were obtained through natural self-pollination of progeny derived from crosses between WT (female) and *cpm/cpm* (male) plants.

The large indels in the rps19 and psbA amplicons were detected in *cpm* seedlings from the F_12_ generation, originating from two distinct F_3_ plants derived from a common F_2_ ancestor [10]. In contrast, the large indels in the rpl33 and rps3 amplicons were identified in F_6_ generation *cpm* seedlings, each derived from different F_2_ plants. The specific polymorphisms identified included: a 15 bp insertion in the intergenic region between the *tRNA^His^* and *rps19* genes; a 620 bp deletion in the *psbA* gene; a 79 bp deletion in the intergenic region between *rpl33* and *rps18*; and a 45 bp deletion in the *rps3* gene.

Additionally, we analyzed seedlings from the progeny of the family in which the *psbA* deletion mutant was originally identified.

### 4.2. DNA Isolation

Genomic DNA was isolated from one or two leaves of individual *cpm* and WT seedlings using a modified version of the Dellaporta micromethod [37]. Leaf tissue was homogenized in Dellaporta isolation buffer using a FastPrep^®^-24 Instrument (MP Biomedicals, Santa Ana, CA, USA), followed by chloroform extraction and DNA precipitation. DNA concentrations were quantified using a NanoDrop™ spectrophotometer (Thermo Scientific, Wilmington, NC, USA) and standardized to 80 ng/µL for downstream applications.

### 4.3. CJE Digestion and Electrophoresis of Amplicons

The four amplicons containing the large indels (see Section 4.1, Plant Material) were amplified using two approaches: (1) using *cpm* DNA mixed with WT DNA to assess homoplastomy, and (2) using *cpm* DNA alone to determine heteroplastomy. Following PCR amplification, a denaturation and slow re-annealing step was performed to promote the formation of heteroduplexes.

Subsequently, the amplicons were digested with CJE as described by Till et al. [38], and analyzed by electrophoresis on 3% non-denaturing polyacrylamide gels [10]. For comparison, undigested amplicons were also subjected to electrophoresis under the same conditions. CJE digestions were confirmed by visualization on agarose gels and by capillary electrophoresis using the Fragment Analyzer™ system (Agilent Technologies, Santa Clara, CA, USA).

### 4.4. Sequencing of the Msh1 Gene Region Carrying the Premature Stop Codon Mutation in the Seedlings with Large Indels

A region of the *Msh1* gene was amplified and sequenced in the four large indel mutants to verify the existence of the stop codon mutation. PCR reaction was performed in a final volume of 10 µL using 40 ng of genomic DNA, 2× Platinum Super Fi II PCR Master Mix (Invitrogen, Carlsbad, CA, USA), and 0.2 µM of each primer. After denaturation at 94 °C for 3 min, the reaction mixtures were heated to 94 °C for 30 s, 60 °C for 1 min, and 72 °C for 1 min in 35 cycles, and 10 min at 72 °C. A 655 bp fragment was amplified using primers Hvmsh1_914F 5’-ACCAGGCAATATCTTCATCGGA-3’ and Hvmsh1_914R 5’-ACCATTCTTCCCCAACCCTTC-3’. PCR products were purified with Exo-CIP™ Rapid PCR Cleanup Kit (New England Biolabs Inc., Ipswich, MA, USA) before sequencing.

### 4.5. Plastome Sequencing and Bioinformatic Analysis

Plastome sequences of the four *cpm* seedlings carrying large indels, along with the WT control, were obtained using a long-amplicon sequencing strategy. This approach was selected over direct chloroplast DNA (cpDNA) isolation due to the limited tissue available from individual plants, which was insufficient for chloroplast or cpDNA extraction.

Primers were designed using Primer3 version 4.1.0 software (Table S6) to amplify 14 overlapping fragments (6–14 kb each, with ~1 kb overlap) covering the entire 136 kb barley chloroplast genome (reference: GenBank NC_008590.1). PCR amplification was performed using LongAmp^®^ Taq DNA Polymerase (New England Biolabs Inc.) following the manufacturer’s protocol. The PCR reactions were performed in a final volume of 25 µL using 80 ng of genomic DNA, 1× Taq buffer, 300 µM dNTPs, 0.4 µM of each primer, and 2.5 units LongAmp Taq DNA Polymerase 5 units/50 µL PCR. After denaturation at 94 °C for 30 s, the reaction mixtures were heated to 94 °C for 30 s, 55–60 °C (depending on the amplicon) for 30 s, and 65 °C for 12 min in 30 cycles, and 10 min at 65 °C. Amplicons were purified using the Exo-CIP™ Rapid PCR Cleanup Kit (New England Biolabs Inc.) and quantified with a Qubit™ fluorometer using the Qubit dsDNA BR Assay Kit (Thermo Fisher Scientific, Waltham, MA, USA).

The 14 amplicons from each plant were normalized to equal concentrations and pooled to generate a single sample per plant for Illumina sequencing. Libraries were prepared using the Nextera XT DNA Sample Preparation Kit (Thermo Fisher Scientific), following the manufacturer’s instructions. DNA was diluted to 0.5 ng/µL, enzymatically fragmented to ~500 bp, and subjected to adapter and index incorporation. After two rounds of library cleanup and normalization, paired-end sequencing (2 × 250 bp) was performed on a MiSeq^®^ platform (Illumina, San Diego, CA, USA) at Unidad de Genómica, IABIMO-IB (INTA-CONICET), Hurlingham, Buenos Aires, Argentina.

Raw reads were quality-checked using FASTQC after trimming low-quality sequences with TRIMMOMATIC. Reads were mapped to the reference genome (NC_008590.1) using Bowtie2 and BWA-MEM, and coverage was assessed using BAM coverage plots. Variant calling for SNPs and indels was performed using Snippy and VarScan, based on BAM file pileups. De novo plastome assemblies were generated using SPAdes and Shovill. All analyses were conducted on the Galaxy platform version 25.0.3.dev0 [39].

Assembled scaffolds were aligned to the reference genome using mVISTA [40,41], and alignments were visually inspected with the Integrative Genomics Viewer (IGV) version 2.3.91 [42]. Genetic variants were annotated and classified based on their predicted effects on annotated genes. Additionally, Sanger sequencing was used to validate the four loci containing large indels, and these sequences were aligned with the WT and reference sequences (NC_008590.1) using Clustal Omega (v1.2.4). The RPS3 protein sequence from WT and mutant barley was also compared with orthologous sequences from other species.

The barley plastome figure showing cpTILLING amplicons and polymorphisms (Figure 6) was done with OGDRAW online software version 1.3.1 [43] and Vector NTI 10.0 Software (Thermo Fisher Scientific, Waltham, MA, USA).

### 4.6. Thylakoid Membrane Isolation and Immunoblotting

Thylakoid membranes were isolated following the protocol described by Guiamet et al. [44]. Equal amounts of thylakoid proteins, normalized based on chlorophyll content, were solubilized and separated by SDS-PAGE using 13% (*w*/*v*) polyacrylamide gels. Following electrophoresis, proteins were transferred to nitrocellulose membranes for immunoblot analysis.

Immunodetection was performed using rabbit polyclonal antibodies specific to various photosynthetic proteins. These included antibodies against PSI subunits A/B, D, E, and L (generously provided by H.V. Scheller, The Royal Veterinary and Agricultural University, Copenhagen, Denmark); LHCB1, a light-harvesting complex protein of PSII (provided by J.J. Guiamet, INFIVE, Universidad Nacional de La Plata, La Plata, Argentina); and the PSII core proteins D1 and D2 (provided by A. Barkan, University of Oregon, Eugene, OR, USA). Detection was carried out using horseradish peroxidase-conjugated goat anti-rabbit secondary antibodies and the SuperSignal™ West Dura Extended Duration Substrate (Thermo Fisher Scientific, Waltham, MA, USA), following the manufacturer’s instructions.

## 5. Conclusions

**Figure 8:**
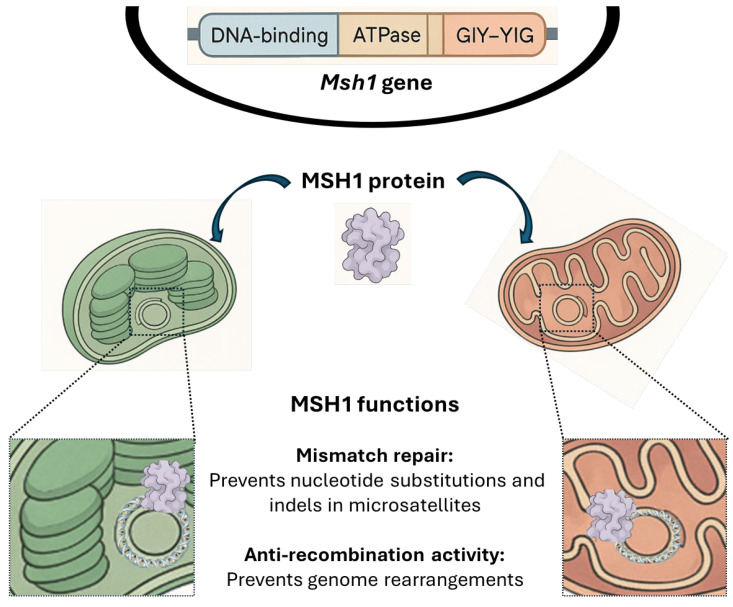
A general scheme of MSH1 protein showing its localization and functions in plants. At the top, the *Msh1* gene in the nucleus, highlighting its functional domains: DNA-binding, ATPase, and GIY-YIG endonuclease. Subcellular localization of MSH1 is shown, indicating its presence in chloroplasts (**left**) and mitochondria (**right**) and its interaction with the DNA molecules of both organelles. The bottom panel summarizes the main functions of MSH1: mismatch repair, which prevents nucleotide substitutions and indels in microsatellites, and anti-recombination activity, which suppresses genome rearrangements. Part of the figure was generated with the assistance of the ClickUp Brain artificial intelligence tool [45].

The identification of short perfect or imperfect direct repeats flanking all four large indels in the plastome of *cpm* seedlings supports the hypothesis that these structural variants may result from elevated rates of illegitimate recombination between short homologous sequences. This mechanism was previously proposed to explain the high frequency of polymorphisms observed in the *rpl23* gene and its pseudogene in *cpm* seedlings. Together with the increased frequency of nucleotide substitutions and small indels in microsatellite regions—reported both in earlier studies and confirmed in this work—these findings are consistent with the hypothesis that the *Cpm* nuclear gene encodes a defective MSH1 protein. The loss of MSH1-mediated surveillance compromises plastome stability, leading to the accumulation of structural and sequence-level mutations observed in *cpm* barley seedlings. A schematic functional model of the MSH1 protein is shown in Figure 8.

## Figures and Tables

**Table 1:** Direct repeats flanking large indels in rps19, psbA, rpl33, and rps3 amplicons. Repeat sequences labeled as “1” and “2” correspond to the repeats located upstream (left) and downstream (right) of each indel, respectively. For indels located within coding regions, the positions of the deleted bases are indicated in brackets, based on the corresponding gene sequence. The insertion in the *tRNA^His^*–*rps19* intergenic region represents a duplication of the sequence TAGAATAATGATCAA.

Large Indel	Reference Genome Position	Plastome Region	Indel Length (bp)	Alignment of Direct Repeats
Insertion in *tRNA^His^*–*rps19*	81544	rps19 intron IRa	15	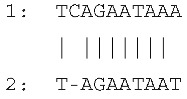
Deletion in *psbA*	845	*psbA* gene LSC	620 (217–836)	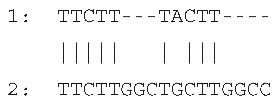
Deletion in *rpl33*–*rps18*	66047	Intergenic region LSC	79	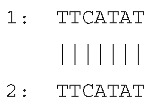
Deletion in *rps3*	80340	*rps3* gene LSC	45 (188–232)	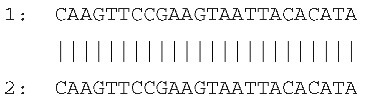

## Data Availability

Data is contained within the article and Supplementary Materials.

## Supplementary Materials

The following supporting information can be downloaded at: https://www.mdpi.com/article/10.3390/ijms26178644/s1.
