# Supplementary material for: Four Large Indels in Barley Chloroplast Mutator (cpm) Seedlings Reinforce the Hypothesis of a Malfunction in the MMR System
Source: Int J Mol Sci. 2025 Sep 5;26(17):8644. doi: 10.3390/ijms26178644 (PMC12429641; doi:10.3390/ijms26178644)
Supplement: Supplementary file 1 [file ijms-26-08644-s001.zip › Supplementary Material Figures.pdf]

Supplementary Figures

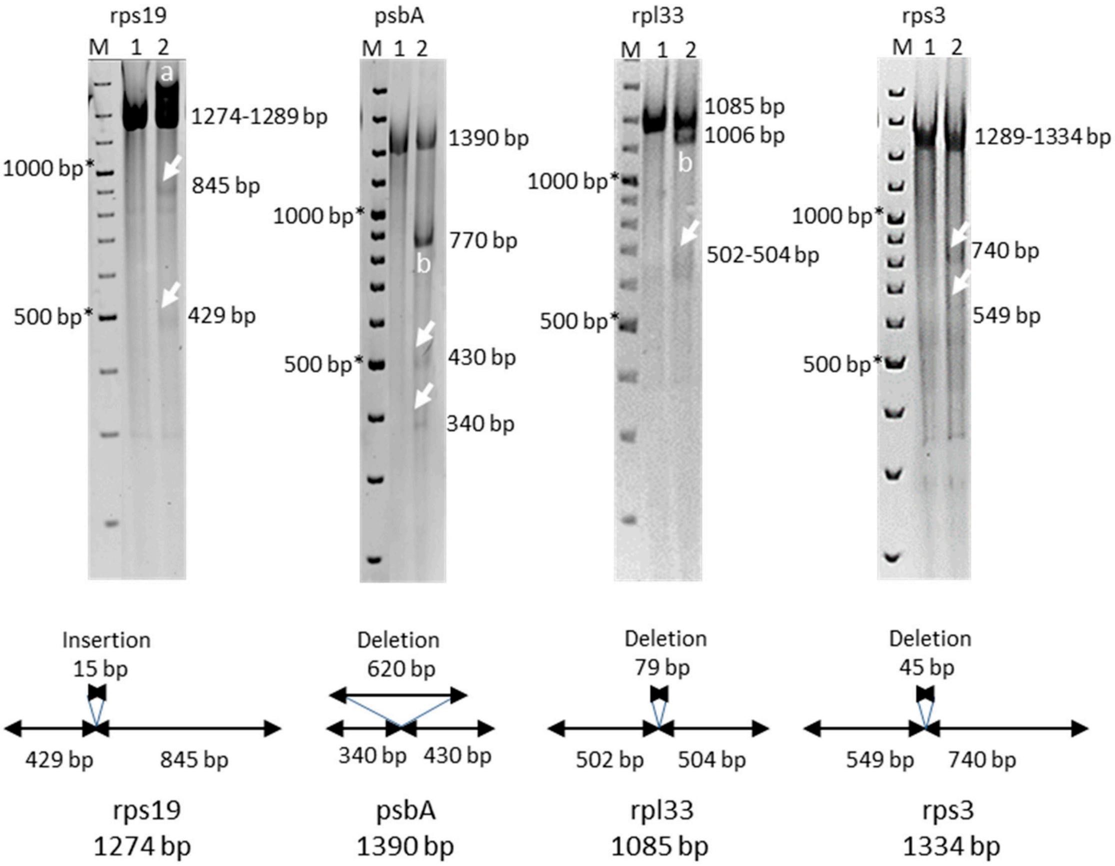

Figure S1 Celery juice extract (CJE) digestion of PCR amplicons containing large indels: rps19, rpl33, psbA and rps3. M: molecular weight marker (100 bp ladder); Lane 1: *cpm* seedling without the indel (wild-type amplicon); Lane 2: *cpm* seedling carrying the corresponding large indel. Arrows indicate digestion products resulting from heteroduplex cleavage. a: secondary amplification product larger than the expected amplicon size; b: secondary amplification product smaller than the expected amplicon size.

Note: Migration of some samples appears slower than the molecular weight marker, likely due to components present in the CJE digestion mixture.

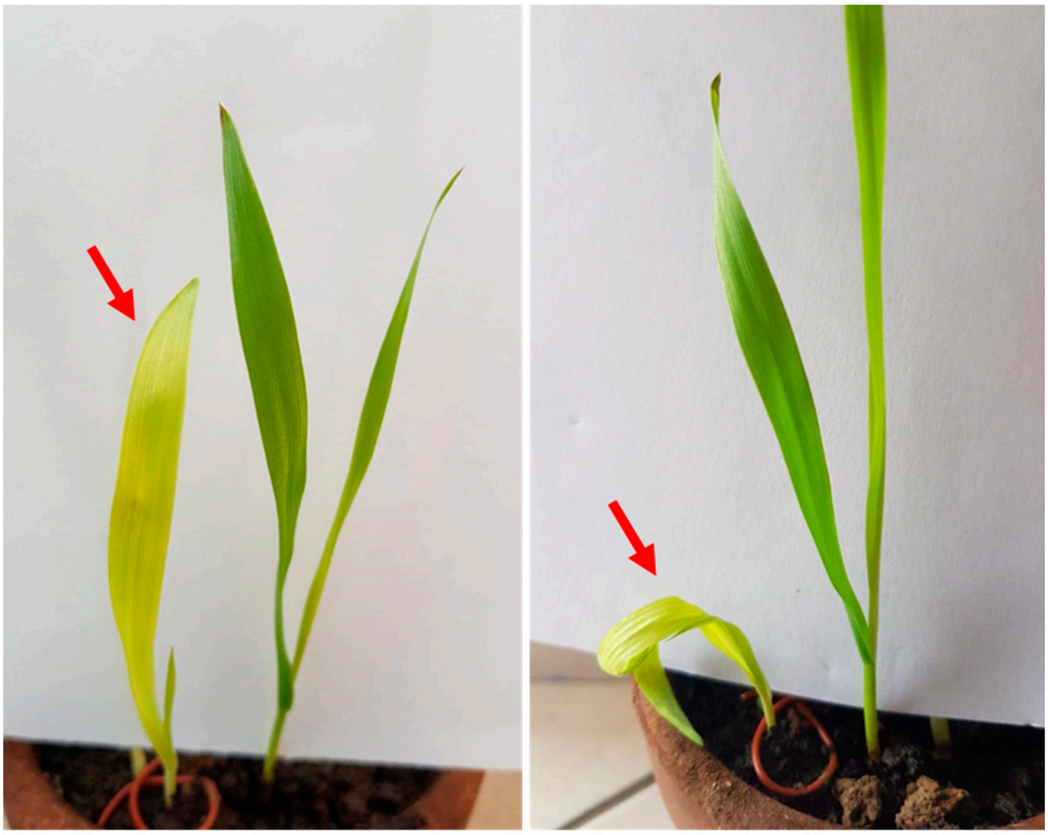

1 2

Figure S2 Barley seedling carrying a homoplastomic 620 bp deletion in the *psbA* gene, exhibiting a *viridis* (pale green) phenotype (indicated by red arrows), alongside phenotypically normal green siblings. Images were taken at two developmental stages: (1) 16 days and (2) 19 days after sowing.

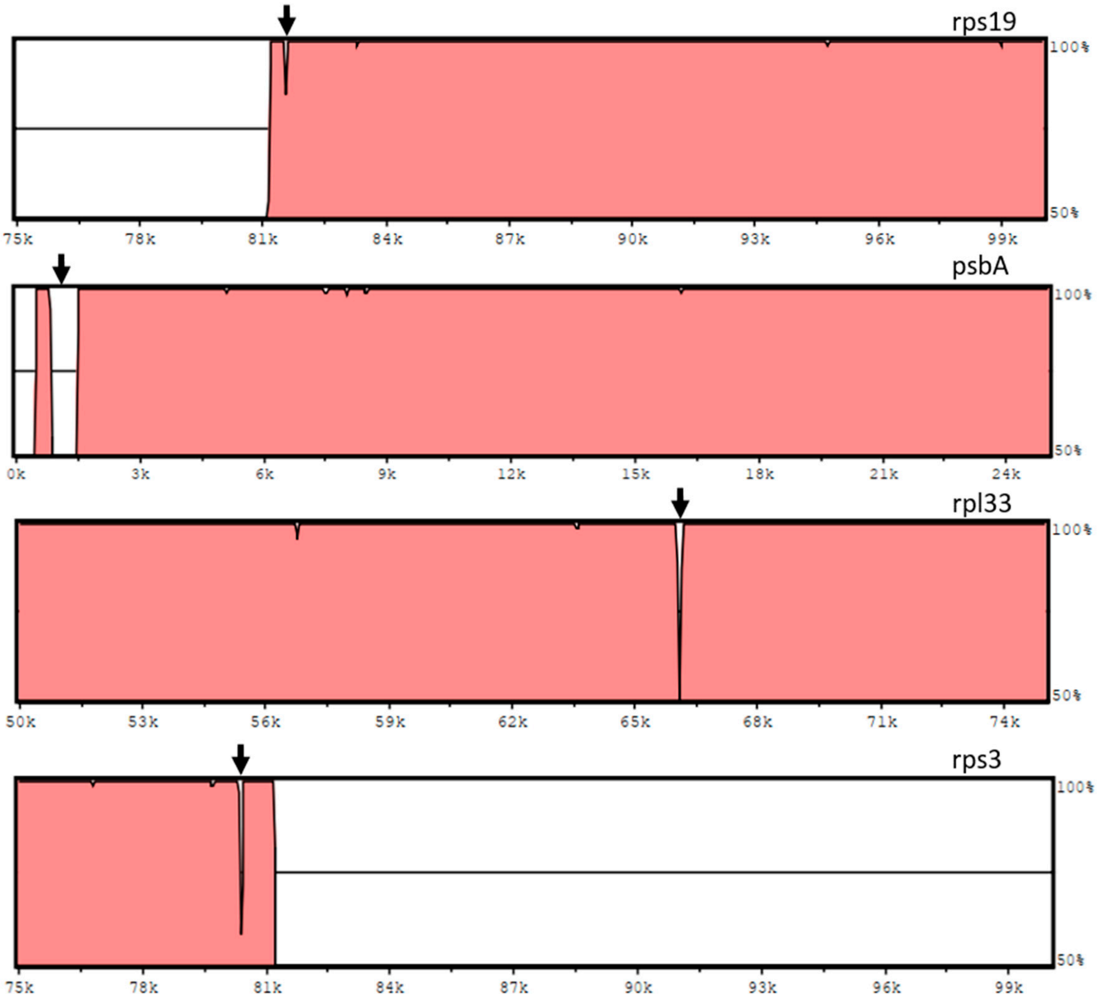

Figure S3 Alignment of de novo assembled scaffolds from barley seedlings carrying large indels in the rps19, psbA, rpl33, and rps3 amplicons (upper x-axis) with the barley reference chloroplast genome (GenBank accession NC_008590.1; lower x-axis). Red-colored regions indicate high sequence identity between the assembled scaffolds and the reference genome. Black arrows mark the positions of the four large indels, located at nucleotide positions 81544 (rps19), 845 (psbA), 66047 (rpl33), and 80340 (rps3) in the reference genome.
