# Supplementary material for: Four Large Indels in Barley Chloroplast Mutator (cpm) Seedlings Reinforce the Hypothesis of a Malfunction in the MMR System
Source: Int J Mol Sci. 2025 Sep 5;26(17):8644. doi: 10.3390/ijms26178644 (PMC12429641; doi:10.3390/ijms26178644)
Supplement: Supplementary file 1 [file ijms-26-08644-s001.zip › Supplementary Material Tables.pdf]

Table S1 Polymorphisms identified in the seedling carrying a large insertion within the rps19 amplicon, based on mapping results from plastome long amplicon sequencing. The 15 bp insertion located in the intergenic region between the *tRNA*^{His} and *rps19* genes was also confirmed by de novo assembly. Superscript **a** denotes polymorphisms detected by CJE digestion, while **b** indicates polymorphisms not detected by CJE digestion during the cpTILLING screening [10,11].

Reference genome position	Type of polymorphism	Reference sequence	Mutant sequence	Homo-plastomic	Hetero-plastomic	Plastome region	Mutation effect	Microsatellite in the reference sequence
7497	Deletion	CA	C		X	Intergenic (LSC)		11 A
15465	Deletion	TA	T		X	Intergenic (LSC)		10 A
16106	Deletion	AT	A		X	Intergenic (LSC)		9 T
21264	Substitution	A	G	X		*rpoB* (LSC)	missense	
29940	Deletion	TA	T		X	*rpoC2* (LSC)	frameshift	12 A
42029	Insertion	C	CT		X	Intergenic (LSC)		9 T
44397	Insertion	C	CA		X	*ycf3* intron 1 (LSC)[b]		11 A
45023	Substitution	T	C	X		Intergenic (LSC)		
47090	Insertion	C	CA		X	Intergenic (LSC)		10 A
64480	Deletion	AT	A		X	Intergenic (LSC)		13 T
71976	Insertion	C	CA		X	*petB* intron 1 (LSC)		8 A

77222	Substitution	A	G	X		Intergenic (LSC)		
79696	Substitution	T	G	X		*rpl16* intron 1 (LSC)[b]		
81544	Insertion	A	ATAGAATAATGATCAA		X	*rps19* intron (IRa)		
83307	Substitution	T	C	X		*rpl23* (IRa)[a]	missense	
94756	Insertion	C	CG		X	Intergenic trans-splicing *rps12* (IRa)		9 G
98978	Substitution	C	T	X		*23S rRNA* (IRa)[a]		
109178	Substitution	C	T	X		*ndhD* (SSC)	missense	
119156	Substitution	G	A	X		*23S rRNA* (IRb)[a]		
123368	Insertion	G	GC		X	Intergenic trans-splicing *rps12* (IRb)		9 C
134827	Substitution	A	G	X		*rpl23* (IRb)[a]	missense	

Table S2 Polymorphisms identified in the seedling carrying a large deletion within the psbA amplicon, based on mapping results from plastome long amplicon sequencing. A 620 bp deletion within the *psbA* gene was also confirmed by de novo assembly. Superscript **a** denotes polymorphisms detected by CJE digestion, while **b** indicates polymorphisms not detected by CJE digestion during the cpTILLING screening [10,11].

Reference genome position	Type of polymorphism	Reference sequence	Mutant sequence	Homo-plastomic	Hetero-plastomic	Plastome region	Mutation effect	Microsatellite in the reference sequence
5105	Substitution	A	G	X		*rps16* (LSC)[b]	silent	
7497	Deletion	CA	C		X	Intergenic (LSC)		11 A
7983	Deletion	ATT	A		X	Intergenic (LSC)		12 T
16106	Deletion	AT	A		X	Intergenic (LSC)		9 T
29940	Deletion	TA	T		X	*rpoC2* (LSC)	frameshift	12 A
30348	Insertion	G	GA		X	Intergenic (LSC)[b]		9 A
32115	Insertion	A	ATT		X	Intergenic (LSC)		10 T
48023	Insertion	A	AC		X	Intergenic (LSC)		9 C
56759	Substitution	T	G	X		*rpl23* pseudogene (LSC)[a]		
56762	Substitution	A	G	X		*rpl23* pseudogene (LSC)[a]		

56776	Substitution and insertion	A	TG	X		*rpl23* pseudogene (LSC)[a]		
56846	Substitution	G	A	X		*rpl23* pseudogene (LSC)[b]		
63576	Deletion	CT	C		X	Intergenic (LSC)		10 T
64480	Insertion	A	AT		X	Intergenic (LSC)		13 T
70757	Substitution	T	C	X		*psbB* (LSC)	silent	
76534	Deletion	CT	C	X		Intergenic trans-splicing *rps12* (LSC)[b]		9 T
76784	Insertion	G	GA		X	Intergenic trans-splicing *rps12* (LSC)[b]		10 A
77349	Substitution	A	G	X		*rps8* (LSC)	silent	
79696	Substitution	T	G	X		*rpl16* intron 1 (LSC)[b]		
94756	Insertion	C	CG		X	Intergenic trans-splicing *rps12* (IRa)		9 G
100932	Substitution	T	C	X		Intergenic trans-splicing *rps12* (IRa)		
113394	Substitution	A	G	X		*ndhA* intron 1 (SSC)		
117202	Substitution	A	G	X		Intergenic trans-splicing *rps12* (IRb)		
123368	Insertion	G	GC		X	Intergenic trans-splicing *rps12* (IRb)		9 C

Table S3 Polymorphisms identified in the seedling carrying a large deletion within the rpl33 amplicon, based on mapping results from plastome long amplicon sequencing. The 79 bp deletion in the intergenic region between the *rpl33* and *rps18* genes was also confirmed by de novo assembly. Superscript **a** denotes polymorphisms detected by CJE digestion, while **b** indicates polymorphisms not detected by CJE digestion during the cpTILLING screening [10,11].

Reference genome position	Type of polymorphism	Reference sequence	Mutant sequence	Homo-plastomic	Hetero-plastomic	Plastome region	Mutation effect	Microsatellite in the reference sequence
16106	Deletion	AT	A		X	Intergenic (LSC)		9 T
29940	Deletion	TA	T		X	*rpoC2* (LSC)	frameshift	12 A
77228	Substitution	A	G	X		*rps8* (LSC)	missense	
79696	Substitution	T	G	X		*rpl16* intron 1 (LSC)[b]		
83307	Substitution	T	C		X	*rpl23* (IRa)[a]	missense	
94756	Insertion	C	CG		X	Intergenic trans-splicing *rps12* (IRa)		9 G
123368	Insertion	G	GC		X	Intergenic trans-splicing *rps12* (IRb)		9 C
134827	Substitution	A	G		X	*rpl23* (IRb)[a]	missense	

Table S4 Polymorphisms identified in the seedling carrying a large deletion within the rps3 amplicon, based on mapping results from plastome long amplicon sequencing. The 45 bp deletion within the *rps3* gene was also confirmed by de novo assembly. Superscript **a** denotes polymorphisms detected by CJE digestion, while **b** indicates polymorphisms not detected by CJE digestion during the cpTILLING screening [10,11].

Reference genome position	Type of polymorphism	Reference sequence	Mutant sequence	Homo-plastomic	Hetero-plastomic	Plastome region	Mutation effect	Microsatellite in the reference sequence
16106	Deletion	AT	A		X	Intergenic (LSC)		9 T
56759	Substitution	T	G	X		*rpl23* pseudogene (LSC)[a]		
56762	Substitution	A	G	X		*rpl23* pseudogene (LSC)[a]		
56776	Substitution and insertion	A	TG	X		*rpl23* pseudogene (LSC)[a]		
56846	Substitution	G	A	X		*rpl23* pseudogene (LSC)[b]		
76784	Insertion	G	GA		X	Intergenic trans-splicing *rps12*[b] (LSC)		10 A
79696	Substitution	T	G	X		*rpl16* intron 1 (LSC)[b]		
83307	Substitution	T	C		X	*rpl23* (IRa)[a]	missense	
98702	Deletion	CG	C		X	*23S rRNA* (IRa)[b]		7 G

108957	Substitution	T	C	X		*ndhD* (SSC)	silent	
119424	Deletion	GC	G		X	*23S rRNA* (IRb)[b]		7 C
134827	Substitution	A	G		X	*rpl23* (IRb)[a]	missense	

Table S5 Polymorphisms identified in more than one *cpm* mutant carrying large indels, based on mapping results from plastome long amplicon sequencing.

Reference genome position	Type of polymorphism	Reference sequence	Mutant sequence	Homo-plastomic	Hetero-plastomic	Plastome region	Mutation effect	Microsatellite in the reference sequence	Present in *cpm* mutants
7497	Deletion	CA	C		X	Intergenic (LSC)		11 A	*psbA* and *rps19*
16106	Deletion	AT	A		X	Intergenic (LSC)		9 T	The four mutants and the control
29940	Deletion	TA	T		X	*rpoC2* (LSC)	frameshift	12 A	*psbA*, *rps19* and *rpl33*
56759	Substitution	T	G	X		rpl23 pseudogene (LSC)			*psbA* and *rps3*
56762	Substitution	A	G	X		rpl23 pseudogene (LSC)			*psbA* and *rps3*
56776	Substitution and insertion	A	TG	X		rpl23 pseudogene (LSC)			*psbA* and *rps3*
56846	Substitution	G	A	X		rpl23 pseudogene (LSC)			*psbA* and *rps3*

64480	Deletion	AT	A		X	Intergenic (LSC)		13 T	*psbA* and *rps19*
76784	Insertion	G	GA		X	Intergenic trans-splicing rps12 (LSC)		10 A	*psbA* and *rps3*
79696	Substitution	T	G	X		rpl16 intron 1 (LSC)[b]			The four mutants and the control
83307	Substitution	T	C		X	rpl23 (IRa)	missense		*rpl33*, *rps3* and *rps19*
94756	Insertion	C	CG		X	Intergenic trans-splicing rps12 (IRa)		9 G	*psbA*, *rps19* and *rpl33*
123368	Insertion	G	GC		X	Intergenic trans-splicing rps12 (IRb)		9 C	*psbA*, *rps19* and *rpl33*
134827	Substitution	A	G		X	rpl23 (IRb)	missense		*rpl33*, *rps3* and *rps19*

Table S6 Primers used for amplifying the chloroplast genome of *Hordeum vulgare cpm* seedlings with long PCR.

AMPLICON	PRIMER F (5' - 3')	Tm	PRIMER R (5' - 3')	Tm	PCR product (bp)
1	GCTACATCCGCCCCTTATCC	60.04	CCATACAAATGCACGGCGAG	59.97	10900
2	GGCTCTGAACTTACGTGCCT	60.04	TTCCCTCGTTTCCATTCCGG	60.04	10084
3	AACTTTAGGTCCGAGCAGCA	59.31	CCGATTAATTCCCCAGGCGA	59.89	10316
4	CGGCATCCAACTGTTCTCGAA	60.94	GCTTAGCTCAGGACCCCACTA	60.97	10823
5	ACTCGAATCAGGGTTTTGGGC	60.89	ATCCGCGCCTTAAACGTATCC	60.87	10008
6	TGTGCTAAGTCATGTGCGCGATACA	65,03	TTCCGTAGAAAGACATCACGACCCC	64.29	10127
7	TACTAACAGGACTTCGCGGC	59.83	ATAAACTCCCCAGGCGGTTG	60.03	11272
8	TCGTATTGGCTGTCTTGCGT	60.04	CGAGTCATAGGAGCTGCTGG	59.97	10023
9	TTTCAAAATAGGAAGGGCGAGGTA	60.02	AAGTTGCCAGAGTACGATTAACCT	60.02	13449
10	CTGCAGTACCTCGACGTGACATGAG	65.03	ACCTCTTGCTTGCTTCTGGTCCAAA	64.89	13987
11	GTCGACGTTTGCTGGCTTATTTGGC	65.45	TAGCAGCTTGGCCAATTACACGGAA	65.02	6963
12	CTGCGACAAAACCACTCATTCCTGG	64.47	CCCTCAACACCCATCAATGCATGGT	65.58	7240
13	GGCCAGCATATTCAGGACCA	59.82	ATGCAAGTCGAACGGGAAGT	59.97	11145
14	GGTCTCTAATCCCATTTGCTCCCCT	63.95	ACGAACGTAATGCTCACAACTTCCC	63.73	12444
